# A multiscale and multiparametric approach for modeling the progression of oral cancer

**DOI:** 10.1186/1472-6947-12-136

**Published:** 2012-11-22

**Authors:** Konstantinos P Exarchos, Yorgos Goletsis, Dimitrios I Fotiadis

**Affiliations:** 1 Unit of Medical Technology and Intelligent Information Systems, Department of Materials Science and Engineering, University of Ioannina, GR45110, Ioannina, Greece; 2 Foundation for Research and Technology - Hellas, Institute of Molecular Biology and Biotechnology, Department of Biomedical Research, GR45110, Ioannina, Greece; 3 Department of Economics, University of Ioannina, GR45110, Ioannina, Greece

## Abstract

**Background:**

In this work, we propose a multilevel and multiparametric approach in order to model the growth and progression of oral squamous cell carcinoma (OSCC) after remission. OSCC constitutes the major neoplasm of the head and neck region, exhibiting a quite aggressive nature, often leading to unfavorable prognosis.

**Methods:**

We formulate a Decision Support System assembling a multitude of heterogeneous data sources (clinical, imaging tissue and blood genomic), aiming to capture all manifestations of the disease. Our primary aim is to identify the factors that dictate OSCC progression and subsequently predict potential relapses of the disease. The discrimination potential of each source of data is initially explored separately, and afterwards the individual predictions are combined to yield a consensus decision achieving complete discrimination between patients with and without a disease relapse. Moreover, we collect and analyze gene expression data from circulating blood cells throughout the follow-up period in consecutive time-slices, in order to model the temporal dimension of the disease. For this purpose a Dynamic Bayesian Network (DBN) is employed which is able to capture in a transparent manner the underlying mechanism dictating the disease evolvement, and employ it for monitoring the status and prognosis of the patients after remission.

**Results:**

By feeding as input to the DBN data from the baseline visit we achieve accuracy of 86%, which is further improved to complete discrimination when data from the first follow-up visit are also employed.

**Conclusions:**

Knowing in advance the progression of the disease, i.e. identifying groups of patients with higher/lower risk of reoccurrence, we are able to determine the subsequent treatment protocol in a more personalized manner.

## Background

Oral cancer refers to the cancer that arises in the head and neck region, i.e. in any part of the oral cavity or oropharynx. OSCC constitutes the 8^th^ most frequent neoplasm in humans according to the worldwide cancer incidence ranking, and has been primarily associated with smoking and alcohol consumption [1]. In terms of sex, men face twice the risk of being diagnosed with oral cancer than women [1]. Moreover, sun exposure constitutes a significant risk factor, particularly for the cancer of the lip. There has also been suggested in the literature, that infection with the Human Pappilomavirus (HPV) is associated with oral cancer, especially with occurrences in the back of the mouth (oropharynx, base of tongue, tonsillar pillars and crypt, as well as the tonsils themselves) [2]. Although current advances in treatment protocols [3] have led to high rates of successful eradication of the disease (i.e. a state called remission), a significant percentage, in the range of 25-48% [4], of remittent patients suffer from locoregional relapses, owed to the deeply infiltrative nature of these tumors, as well as the significant potential for occult neck metastasis [5]. The accurate modeling of the disease progression and consequently the timely identification of a potential reoccurrence can provide patient-specific treatment.

In the literature, several studies have identified factors affecting the oral cancer invasion, progression and metastasis, both from a clinical and molecular perspective; yet they still remain limited in number and efficacy, leading to unsatisfactory results [6]. Specifically, [7,8] derive a gene expression profile in order to diagnose lymph node metastasis originating from a primary head and neck carcinoma; similarly, in [9], future metastases of head and neck carcinoma are predicted. In [10-12], the progression of tongue carcinoma is studied, and a subset of genes is identified, able to predict potential metastasis of the primary tumor is in the lymph nodes. Recently Reis et al. [13] have performed a meta-analysis based on five publicly available microarray datasets, and identified a four-gene signature that is of prognostic value for oral cancer reoccurrence. However, it should be noted that all the aforementioned approaches do not take into account the temporal dimension of the disease and its actual evolution over time. Other approaches identified in the broader field of biomedical engineering deal with pairs of consecutive time-slices rather than representing the follow-up as a whole [14].

The proposed approach encompasses in a complementary manner a multitude of heterogeneous data, varying in scale and dimension, therefore, "framing" all possible manifestations of the disease, from a clinical, imaging and genomic point of view. Among the aims of this work is to identify a limited subset of factors that are highly correlated with oral cancer progression, thus, formulating the disease profile. Based on this profile, we are able to calculate the risk of relapse for each patient and subsequently discriminate the patients in high and low risk groups. Moreover, information derived from time-varying parameters (i.e. gene expression from circulating blood cells) is employed in order to model the evolvement of the disease over time, and capture the temporal dimension of the disease as well. The outcome of this analysis is the representation of the disease mechanism which is subsequently used in order to conjecture if, when and why a patient is prone to suffer a relapse of the disease. Having a timely and accurate estimation of the relapse probability can be proven quite helpful towards determining the most proper treatment for a specific patient; i.e. patients in high risk can be monitored more intensely, whereas, patients in low risk are subject to less aggressive treatment.

In the sections that follow, we lay out our approach, which is organized in two types of analyses, namely: i) Baseline Data Analysis, which involves the analysis of data collected during the baseline visit of each patient, and are used to stratify the patient either at high or low risk of developing a relapse; ii) Disease Evolution Monitoring, which employs data varying over the follow-up period, i.e. gene expression from circulating blood cells, in order to assess the relapse probability coupled with the approximate timing that this relapse is more likely to occur.

## Methods

### Clinical scenario

In the current study, we consider 86 patients [NeoMark project, FP7-ICT-2007-224483, ICT enabled prediction of cancer reoccurrence - D6.1: Research protocol] that have been enrolled from three major clinical centers residing in Italy (University Hospital of Parma and National Cancer Center Regina Elena) and Spain (MD Anderson Cancer Center). The research for this paper has been conducted in compliance with the Helsinki Declaration; the protocol of the study has been edited in compliance to the Good Clinical Practice and approved by the following Ethical Committees: Comitato Etico Unico per la Provincia di Parma and Ethical Committee of Centro Médico Oncológico MD Anderson España. Written, informed consent was obtained from each patient prior to study participation. All patients have been diagnosed with OSCC and have reached remission, after successful therapeutic intervention (i.e. surgery, chemo/radio-therapy). Thereafter, we collect clinical, imaging and gene expression data, both from the primary tumor as well as from circulating blood cells, at the baseline state of the patient. The clinical data contain standard measurements and laboratory markers from the patient's medical record as well as pathology and risk factor data referring to the organism as a whole (Table 1).

**Table 1:** Clinical features examined in this work

Ecog status	Mobile prosthesis	Body mass index (BMI)	Grade of differentiation
Weight	Dental Cusps	Substance Exposition	Surgical Margins
Height	Galvanic Current	Precancerous Lesions	Martinez-Gimeno Score
Diabetes	Oral Hygiene	**Duration**	Anneroths Mod Score
Allergies	Infection	Immunosuppressor Treatments Presence	D2_40Stain
Cholesterol	**Type Of Infection**	**Immuno Duration**	P53_STAIN
Hypertension	Physical Agents	**Immuno Type**	P16Ink4aStain
Family History Of Malignance	**Type Of Physical Agent**	Tumor Maximum Diameter	EGFR Stain
Smoker	Diet Deficit	Tumor Thickness	CyclinD1Stain
Smoking Habits	**Fe Haematic Concentration**	Depth Of Invasion	Ki67Stain
Quantity Per Day	Plummer Vinson	Basaloid Features	HPV_DNA
Smoking For	**Hb Haematic Concentration**	Lympho Plasmacytic Rection	T Staging
Ex Smoker	**B12 Vitamins Haematic Concentration**	Lympho Plasmacytic Invasion	N Staging
**Quitted Smoking**	**A Vitamins Haematic Concentration**	Perineural Invasion	M Staging
Alcohol	**E Vitamins Haematic Concentration**	Degree Of Cells Keratinisation	
Drinking Habits	**Folati**	Nuclear Pleomorphism	
Mechanical Trauma	Eating Habits	Number of Mitoses per 10HPF	

The imaging data contain image-extracted information after derived after processing the CT and MR imaging modalities of the primary tumor mass and adjacent lymph nodes of the head and neck area (Table 2). As for the genomic data, these include gene expression information both from the primary cancerous tissue specimen as well as from circulating blood cells. During the follow-up period and for an 24-month time span [4], blood genomic data are further gathered from the patient regularly, during scheduled visits planned in consecutive time intervals.

**Table 2:** Imaging features employed in this work

Contrast take-up rate	Necrosis	Side of lymph nodes
Minor Axis Bigger than 10mm	Central Necrosis	Side Relative to Tumor
Extra Nodal Spreading	Bone Infiltration	Cluster
Shape Deviation	Carotid Infiltration	Number of Lymph Nodes
Texture	Cuteneous Invasion	Number of Lymph Nodes Bigger than 3
Water Content	Site of lymph nodes	

**Figure 1:**
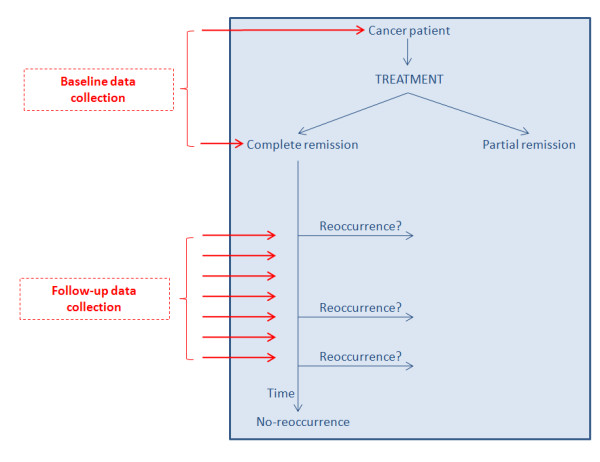
The clinical scenario.

An overview of the employed clinical scenario is depicted in Figure 1. Subsequently, patients are stratified into two groups, namely relapsers and non-relapsers based on the occurrence or not of a potential disease relapse during the follow-up. More specifically, 26 patients have already suffered a relapse, whereas the remaining 60 are still disease-free and constitute the control group of patients in our study.

### Baseline data analysis

**Figure 2:**
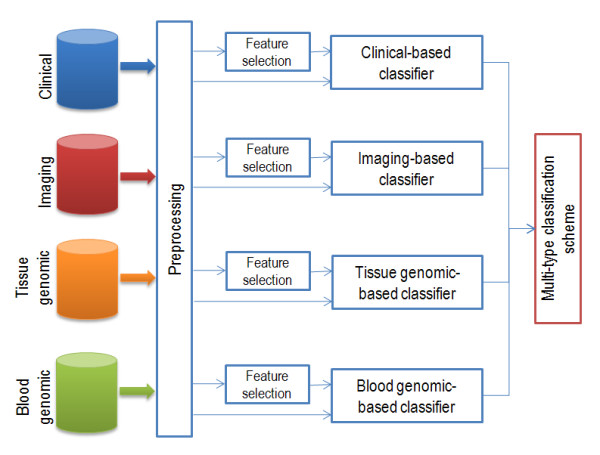
Baseline data analysis flowchart.

The aim of the Baseline Data Analysis is to exploit the information from the baseline data of patients in order to compute the probability of a potential relapse, and consequently stratify the patients into high and low risk groups according to the reoccurrence probability. For this purpose, four sources of data have been utilized; specifically clinical, imaging, tissue genomic and blood genomic data, aiming to identify the most significant features that are effective in detecting a potential relapse. Initially, each source of data is treated independently, subject to the steps shown in Figure 2, in order to estimate the probability of a potential relapse; next, a consensus scheme is implemented that combines the individual predictions in a complementary manner, using a weighted voting algorithm.

As shown in Figure 2, the flow of operations applied upon each source of data includes a series of preprocessing steps; next, the resulting input vector is subject to feature selection in order to maintain the most discriminatory features, which are subsequently fed into a classification algorithm that assigns each patient either as high risk or low risk in terms of the calculated relapse probability. Eventually, a multi-type classification scheme combines all four individual single-classifier decisions, in order to yield an overall outcome. The preprocessing steps, the feature selection schemes as well as the classification algorithms have been invoked through the Weka Machine Learning software [15].

#### Preprocessing

During this step, we utilize a series of preprocessing steps in order to enhance the quality of the input. Specifically, features with high percentage (>90%) of missing values are omitted from our analysis, whereas the values of the features with less percentage of missing values are imputed with the modes and means, in the case of nominal and numeric features, respectively. Another important issue present in our dataset is that the enrolled patients are unevenly distributed in the classes of relapsers and non-relapsers, resulting in considerable class imbalance. For this purpose, we employ the Synthetic Minority Oversampling Technique (SMOTE) [16], which utilizes a k-NN approach in order to oversample the minority class. It should be noted that SMOTE neither discards potentially useful samples nor merely replicates existing samples, therefore, posing an advantageous solution compared to sampling-based approaches. On the contrary, undersampling of the majority class may lead to loss of significant information, whereas, oversampling of the minority class does not add any new information and may duplicate possibly noisy or even erroneous instances.

Especially for the case of genomic data (both tissue and blood ones), the initial feature vector consisting of 45,015 gene expression values, is subject to certain steps in order to enhance the quality of the raw input data, that is subsequently employed for data analysis; to this end, redundant and control genes are removed, as well as genes with low data quality or high percentage of missing values. The outcome is a set of 33,491 high quality genes that are further analyzed in order to procure a limited subset of genes that are mostly differentially expressed between relapsers and non-relapsers. For this purpose we employ the Significance Analysis of Microarrays (SAM) algorithm [17], which performs multiple gene specific t-tests on permutations of the initial dataset, in order to account for the initially enormous number of input genes.

#### Feature selection

As soon as the feature vectors from each source of data are assembled, we either feed them directly as input to the classification algorithms, or employ a feature selection algorithm in order to discard redundant or correlated features and maintain a more informative subset, thus, facilitating the classification task as well. For this purpose two feature selection algorithms have been employed, namely the Correlation-based Feature Subset selection (CFS) [18] and the wrapper algorithm [19] (with the default settings of the Weka software). CFS maintains features that exhibit low correlation among them and high correlation with the class attribute; on the other hand, the wrapper algorithm evaluates all possible feature combinations and retains the best performing subset, tailored to the target classification algorithm.

#### Classification

Next, we examine the performance of six popular classification algorithms [20] towards the discrimination between patients with and without disease relapse; specifically, we employ Bayesian Networks (BN), Naive Bayes (NB), Artificial Neural Networks (ANN), Support Vector Machines (SVM), Decision Trees (DT) and Random Forests (RF).

For evaluation purposes, we employ two techniques, namely 10-fold cross validation and leave-one-patient-out. During 10-fold cross validation the dataset is split into 10 stratified sets, whereby 9/10 are used for training and the remaining 1/10 is used for testing; after a full rotation, the results over the 10 testing sets are averaged in order to procure the overall performance of the algorithm. The leave-one-patient-out technique is quite similar to 10-fold cross validation, where the number of folds is equal to the number of patients in the dataset. Specifically all patients but one are used for training and the remaining one is used for testing in a round-robin manner. The evaluation metrics used to compare the employed classification schemes are: sensitivity, specificity, accuracy, the Kappa statistic, as well as the Area Under Curve (AUC), which is an evaluation index obtained from the Receiver Operating Curve (ROC) analysis.

### Disease evolution monitoring

**Figure 3:**
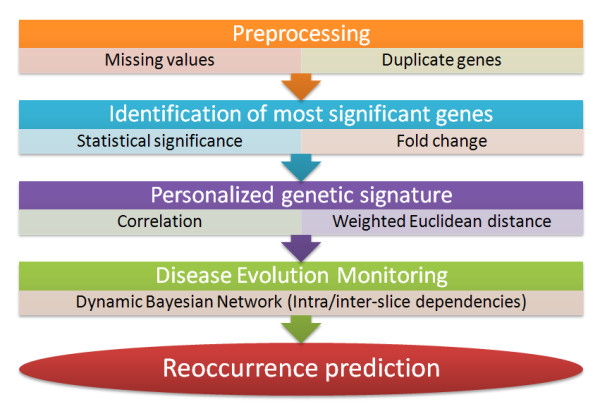
Disease evolution monitoring flowchart.

In the second part of our analysis (i.e. Disease Evolution Monitoring), we aim to predict the probability of a patient to develop a relapse during the follow-up period; hence, we introduce the time dimension and estimate the approximate timing that a relapse is more likely to occur. For this purpose, we employ time-course gene expression data, extracted at predefined time-intervals during the follow-up period from circulating blood cells. Data from 23 patients are analyzed, out of which 11 have already been diagnosed with a disease relapse and the remaining 12 are disease free. The flowchart of the Disease Evolution Monitoring analysis is depicted in Figure 3.

Initially, the gene expression data obtained from 45,015 genes are filtered in order to omit duplicate and control genes as well as the ones that are of low quality; it should be noted that all microarray experiments have been conducted using the same platform, the same array design and the same feature extraction software version in order to exclude unwanted data perturbations other than biological variability. Next, we identify the genes that are mostly differentially expressed between relapsers and non-relapsers; in addition, we extract a personalized gene subset (i.e. Personalized Genetic Signature) aiming to capture patient-specific perturbations of the disease progression within its molecular basis. Both the aforementioned inputs are fed as input to a Dynamic Bayesian Network (DBN) [21] in order to model the disease evolution over the follow-up and predict potential relapses.

#### Identification of the most significant genes

After applying some basic filtering steps upon the gene expression data (i.e. omission of duplicate, control and low-quality genes), we are left with a set of 33,491 genes, that are fed to the next steps of our analysis. The SAM algorithm [17] is subsequently employed, which analyzes differentially unpaired time-course gene expression data between two groups; specifically we perform the Wilcoxon statistical test which identifies those genes that are mostly differentially expressed between the two groups of patients in all time-slices of the follow-up.

#### Personalized Genetic Signature

In addition, we extract a patient-specific genetic indicator denoting the progression of the disease for a specific patient; for each patient we compare the gene expression values before treatment (cancerous profile) and during the first stages of remission (cancer-free profile). The outcome is a limited set of differentially expressed genes representative for each patient, allowing for personalized modeling of the disease evolvement. The expression of these genes from all succeeding follow-up visits is compared in turn with the cancerous and the cancer-free profile, calculating the correlation and the Euclidean distance; these metrics provide, respectively, a qualitative and quantitative measure of the patient's prognosis. In the case of the Euclidean distance a weighted variant is employed which takes into account the significance of each gene in the personalized genetic signature. This weighting factor is proportional to the differential expression of each gene between the cancerous and the cancer-free profile.

#### Dynamic Bayesian Networks

**Figure 4:**
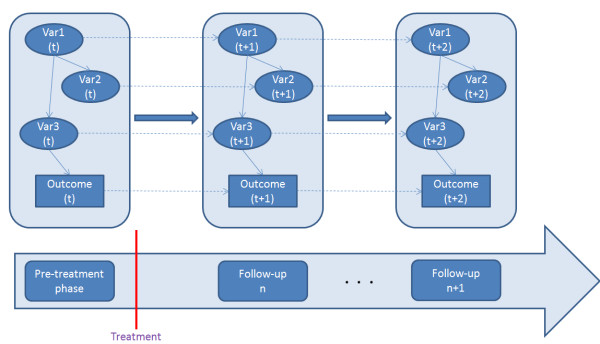
**Provisional architecture of a DBN.** Each VarN in the oval shape corresponds to a specific variable/feature that has been recorded in consecutive time slices.

In the next step of our analysis, we employ a DBN aiming to identify potential relapses of the disease, as well as the approximate time-frame of the relapse. DBNs are basically temporal extensions of Bayesian Networks, as shown in the provisional DBN architecture of Figure 4.

In order to estimate the best performing DBN architecture, i.e. the dependencies among the employed variables within the same time-slice (intra-slice dependencies), as well as across successive time-slices (inter-slice dependencies), we employ two search algorithms, namely the Greedy and the Simulated Annealing. Based on the trained model, we are able to conjecture the values of all variables from future time-slices, including of course the probability of reoccurrence, by using the junction tree algorithm for inferencing from the DBN. In order to formulate and fine-tune the DBN network, as well as for inferencing from the trained model, the miniTUBA system has been used [22]. Due to the relatively limited number of patients available for evaluating the Disease Evolution Monitoring, we employ the leave-one-patient-out technique which is rather suited for making the most out of refined datasets.

## Results

### Baseline data analysis

In the sections that follow, we present the results obtained in our effort to stratify the patients in high and low risk groups based on the reoccurrence probability. Initially, each source of data is treated independently and subsequently all single-classifier decisions are combined using a multi-type classifier. In the first step, each source of data is subject to preprocessing, and then the resulting feature vector is fed to the target classifier either unaltered or after applying certain feature selection algorithms, i.e. CFS and wrapper. As for the actual classification task, we have examined the performance of the following classifiers: Bayesian Networks, Naive Bayes, Artificial Neural Networks, Support Vector Machines, Decision Trees and Random Forests.

#### Clinical-based classification

Table 3 presents the results obtained using solely the clinical data, with all three features selection schemes, i.e. without performing feature selection, using the CFS algorithms and the wrapper algorithm, and feeding subsequently the resulting feature vector as input to a series of classification algorithms.

**Table 3:** Results obtained using the clinical data and all classification schemes

	**Classification algorithm**	**Acc. (%)**	**Se. (%)**	**Sp. (%)**	**Kappa**	**AUC**
**No feature selection**	**BN**	73.7(±8.86)	61.4(±16.04)	86(±10.86)	0.47(±0.18)	0.775(±0.13)
	**NB**	74.6(±15.54)	68.4(±14.72)	80.7(±18.31)	0.49(±0.31)	0.721(±0.15)
	**ANN**	74.6(±15.65)	73.7(±19.78)	75.4(±19.25)	0.49(±0.31)	0.781(±0.12)
	**SVM**	74.6(±11.84)	71.9(±17.93)	77.2(±20.73)	0.49(±0.24)	0.746(±0.12)
	**DT**	81.6(±11.21)	73.7(±14.35)	89.5(±11.91)	0.63(±0.22)	0.826(±0.10)
	**RF**	74.6(±13.98)	68.4(±18.19)	80.7(±16.56)	0.49(±0.28)	0.807(±0.09)
**CFS**	**BN**	73.7(±10.20)	64.9(±15.56)	82.5(±12.18)	0.47(±0.20)	0.776(±0.08)
	**NB**	77.2(±9.73)	68.4(±14.42)	86(±12.15)	0.54(±0.19)	0.783(±0.10)
	**ANN**	71.9(±10.03)	68.4(±14.35)	75.4(±15.07)	0.44(±0.20)	0.736(±0.10)
	**SVM**	78.1(±10.26)	66.7(±17.43)	89.5(±9.09)	0.56(±0.21)	0.781(±0.10)
	**DT**	77.2(±11.88)	66.7(±12.09)	87.7(±15.96)	0.54(±0.24)	0.788(±0.07)
	**RF**	72(±14.12)	64.9(±16.94)	78.9(±16.39)	0.44(±0.28)	0.75(±0.09)
**Wrapper**	**BN**	74.6(±10.62)	66.7(±18.21)	82.5(±8.05)	0.49(±0.22)	0.758(±0.15)
	**NB**	78.1(±10.59)	73.7(±14.56)	82.5(±9.19)	0.56(±0.21)	0.791(±0.09)
	**ANN**	77.2(±14.39)	73.3(±11.09)	80.7(±19.95)	0.54(±0.29)	0.791(±0.13)
	**SVM**	78.1(±11.62)	66.7(±17.43)	89.5(±11.65)	0.56(±0.23)	0.781(±0.12)
	**DT**	83.3(±10.06)	73.7(±14.35)	93(±8.05)	0.67(±0.20)	0.842(±0.09)
	**RF**	75.4(±8.60)	71.9(±12.01)	78.9(±8.05)	0.51(±0.17)	0.826(±0.11)

Acc.: Accuracy; Se.: Sensitivity; Sp.: Sensitivity; AUC: Area Under Curve.

The employment of the CFS algorithm for feature selection maintains the following features as most discriminatory: smoker, tumor thickness, lymphoplasmacytic rection, perineural invasion, num mitoses HPF, surgical margins, p53 stain and N staging. The employment of the wrapper algorithm has pinpointed the following features for each classification algorithm: BN: ecog status, cholesterol, grade differentiation and N staging; NB: allergies, cholesterol, depth invasion, lympphoplasmacytic rection and N staging; ANN: ecog status, cholesterol, depth of invasion and N staging; SVM: ecog status, smoking duration and N staging; DT: depth of invasion, p16ink4a stain and N staging; RF: quantity of cigarettes, galvanic current, eating habits, BMI, depth invasion and N staging.

#### Imaging-based classification

In Table 4 that follows, we present the results obtained using the imaging data and the classification schemes as laid out previously.

**Table 4:** Results obtained using the imaging data and all classification schemes

	**Classification algorithm**	**Acc. (%)**	**Se. (%)**	**Sp. (%)**	**Kappa**	**AUC**
**No feature selection**	**BN**	86.4(±10.48)	77.3(±17.98)	95.5(±9.56)	0.73(±0.21)	0.936(±0.07)
	**NB**	87.5(±11.96)	75(±23.21)	100(±0)	0.75(±0.24)	0.901(±0.12)
	**ANN**	83(±10.65)	81.8(±13.58)	84.1(±18.83)	0.69(±0.21)	0.914(±0.07)
	**SVM**	84.1(±12.15)	81.8(±17.98)	86.4(±19.16)	0.68(±0.24)	0.841(±0.12)
	**DT**	77.3(±17.71)	72.7(±25.41)	81.8(±27.29)	0.55(±0.35)	0.738(±0.19)
	**RF**	83(±8.02)	72.7(±16.70)	93.2(±16.36)	0.66(±0.16)	0.915(±0.11)
**CFS**	**BN**	85.23(±11.05)	81.8(±16.91)	88.6(±15.06)	0.7(±0.22)	0.917(±0.05)
	**NB**	77.3(±6.82)	65.9(±13.12)	88.6(±15.06)	0.55(±0.13)	0.881(±0.07)
	**ANN**	83(±11.72)	84.1(±14.14)	81.8(±11.35)	0.66(±0.23)	0.89(±0.10)
	**SVM**	87.5(±12.25)	84.1(±14.76)	90.9(±14.15)	0.75(±0.24)	0.875(±0.12)
	**DT**	84.1(±11.72)	77.3(±14.42)	90.9(±14.15)	0.68(±0.23)	0.831(±0.13)
	**RF**	83(±11.72)	79.5(±13.58)	86.4(±14.54)	0.66(±0.23)	0.887(±0.11)
**Wrapper**	**BN**	85.2(±12.25)	84.1(±14.76)	86.4(±14.15)	0.7(±0.24)	0.866(±0.11)
	**NB**	90.9(±12.25)	88.6(±14.76)	93.2(±14.15)	0.82(±0.24)	0.89(±0.12)
	**ANN**	89.8(±12.25)	86.4(±14.76)	93.2(±14.15)	0.8(±0.24)	0.854(±0.13)
	**SVM**	86.4(±12.25)	86.4(±14.76)	86.4(±14.15)	0.73(±0.24)	0.864(±0.12)
	**DT**	85.2(±13.64)	86.4(±14.76)	84.1(±25.58)	0.7(±0.28)	0.835(±0.24)
	**RF**	88.6(±12.25)	84.1(±14.76)	93.2(±14.15)	0.77(±0.24)	0.906(±0.13)

The employment of the CFS algorithm for feature selection resulted in a refined feature vector containing the following features: extra-tumor spreading, extra-nodal spreading, texture (lymph node), site (lymph node), side (lymph node) and number of lymph nodes. Afterwards, the employment of the wrapper feature selection algorithm has resulted in the following lists of features for each classification algorithm: BN: extra-tumor spreading and site (lymph node); NB: extra-tumor spreading and site (lymph node); ANN: extra-tumor spreading and site (lymph node); SVM: extra-tumor spreading, floor of the mouth invasion and site (lymph node); DT: extra-tumor spreading, perineural infiltration (tumor), bone infiltration (tumor) and site (lymph node); RF: extra-tumor spreading and site (lymph node).

#### Tissue genomic-based classification

Using the gene expression values acquired from the cancerous tissue, we aim to identify those genes that are mostly differentially expressed between relapsers and non-relapsers; for this purpose we employ the SAM algorithm and set the fold-change threshold to 1.8, thus, yielding a set of 9 genes, shown in Table 5.

**Table 5:** List of the most significant genes as pinpointed by the SAM algorithm

TCAM1	AMDHD1	SLC5A12
SOD2	AY358224	AK026836
FCAR	PHACTR1	RPRM

Subsequently, the retained genes are employed as part of a series of classification schemes in order to discriminate the patients into high and low risk groups, in terms of reoccurrence probability. The results obtained with all employed classification schemes are shown in Table 6.

**Table 6:** Results obtained using the tissue genomic data and all classification schemes

	**Classification algorithm**	**Acc. (%)**	**Se. (%)**	**Sp. (%)**	**Kappa**	**AUC**
**No feature selection**	**BN**	75.8(±15.44)	75(±21.15)	76.7(±19.56)	0.52(±0.31)	0.843(±0.09)
	**NB**	74.2(±10.72)	70(±20.49)	78.3(±13.72)	0.48(±0.21)	0.834(±0.12)
	**ANN**	74.2(±12.70)	83.3(±13.61)	65(±21.44)	0.48(±0.25)	0.834(±0.13)
	**SVM**	74.2(±12.08)	75(±18.00)	73.3(±16.10)	0.48(±0.24)	0.742(±0.12)
	**DT**	69.2(±11.82)	68.3(±21.44)	67(±25.82)	0.38(±0.24)	0.706(±0.14)
	**RF**	80(±8.96)	85(±14.59)	75(±23.90)	0.6(±0.18)	0.856(±0.10)
**CFS**	**BN**	75.8(±14.83)	75(±20.86)	76.7(±17.21)	0.52(±0.30)	0.838(±0.09)
	**NB**	75(±12.91)	75(±17.66)	75(±19.64)	0.5(±0.26)	0.837(±0.10)
	**ANN**	78.3(±10.54)	83.3(±13.15)	73.3(±14.05)	0.57(±0.21)	0.854(±0.13)
	**SVM**	70.1(±9.46)	73.3(±18.00)	68.3(±13.72)	0.42(±0.19)	0.708(±0.09)
	**DT**	72.5(±13.72)	73.3(±16.20)	71.7(±26.59)	0.45(±0.27)	0.724(±0.15)
	**RF**	76.7(±11.92)	80(±17.57)	73.3(±22.50)	0.53(±0.24)	0.845(±0.09)
**Wrapper**	**BN**	74.2(±12.08)	70(±18.92)	78.3(±14.59)	0.48(±0.24)	0.782(±0.10)
	**NB**	73.3(±11.15)	73.3(±18.34)	73.3(±10.54)	0.47(±0.22)	0.833(±0.10)
	**ANN**	73.3(±15.32)	81.7(±16.57)	65(±19.33)	0.47(±0.31)	0.772(±0.12)
	**SVM**	77.5(±9.00)	76.7(±16.57)	78.3(±14.05)	0.55(±0.18)	0.775(±0.09)
	**DT**	62.5(±14.83)	70(±15.32)	55(±17.66)	0.25(±0.20)	0.665(±0.15)
	**RF**	77.5(±13.49)	78.3(±17.21)	76.7(±23.57)	0.55(±0.27)	0.842(±0.13)

The utilization of the CFS algorithm for feature selection, subsequently retains the following features: TCAM, SOD2, AMDHD1, AY358224, PHACTR1, AK026836 and RPRM. Afterwards, the employment of the wrapper algorithm coupled with a different target classification algorithm each time has retained the following features: BN: TCAM1, AMDHD1, AY358224, PHACTR1 and RPRM; NB: TCAM1, SOD2, AMDHD1, PHACTR1 and RPRM; ANN: TCAM1, SOD2, AMDHD1, PHACTR1, SLC5A12, AK026836 and RPRM; SVM: TCAM1, SOD2, FCAR, AMDHD1, AY358224 and PHACTR1; DT: AMDHD1, AY358224, PHACTR1, AK026836 and RPRM; RF: SOD2, FCAR, AMDHD1, AY358224, PHACTR1 and RPRM.

In the literature, there have been identified several genes that are descriptive of the development and the prognosis of oral cancer, therefore, we have additionally integrated these genes with the ones identified in our work, in order to gain enhanced generalization capability and explore the overall discriminative potential of the resulting unified gene set. The literature derived set consists of 28 genes [23,24] whose performance has been evaluated upon the current dataset, using the same methodology as described in the previous sections. Table 7 provides a comparison among the highest results obtained using the genes identified in the current work, the literature extracted genes and the union of the two gene sets.

**Table 7:** Comparison among the most prominent classification schemes

**Source of gene set**	**Classifier**	**Acc (%)**	**Se (%)**	**Sp (%)**	**Kappa statistic**	**AUC**	**# of genes**
**Literature**	RF	78.94(±8.93)	82.5(±11.20)	75.4(±16.56)	0.58(±0.18)	0.841(±0.07)	28
**Current work**	RF	80(±8.96)	85(±14.59)	75(±23.90)	0.6(±0.18)	0.856(±0.10)	9
**Union of literature and current work**	ANN	91.23(±7.23)	94.7(±11.25)	87.7(±13.72)	0.82(±0.14)	0.957(±0.04)	37

It is noteworthy that even though both gene sets perform quite satisfactory, the union of the two gene sets significantly ameliorates the obtained results, enhancing the generalization capability of the classification procedure.

#### Blood genomic-based classification

Same as with the tissue genomic data, for the blood genomic data as well, initially we employ the SAM algorithm in order to identify the genes that are mostly differentially expressed between relapsers and non-relapsers; the obtained gene subset is shown in Table 8.

**Table 8:** List of most significant genes as pinpointed by the SAM algorithm

THC2410448	BM683433	A_24_P942151
A_24_P221960	OXCT2	X58809
THC2399272	A_24_P230388	AL566369
CN391963	A_32_P57247	

The discriminative potential of the gene subset is evaluated either by providing it directly as input to a series of classification algorithms, or by applying certain feature selection algorithms, prior to the classification task. The results obtained when the 11 genes are used for classification without performing feature selection are shown in Table 9.

**Table 9:** Results obtained using the blood genomic data and all classification schemes

	**Classification algorithm**	**Acc. (%)**	**Se. (%)**	**Sp. (%)**	**Kappa**	**AUC**
**No feature selection**	**BN**	87.5(±21.94)	83.3(±42.16)	91.7(±31.62)	0.75(±0.48)	0.965(±0)
	**NB**	91.7(±21.08)	91.7(±31.62)	91.7(±31.62)	0.83(±0.42)	0.986(±0)
	**ANN**	95.8(±15.81)	100(±0)	91.7(±31.62)	0.92(±0.32)	1(±0)
	**SVM**	95.8(±15.81)	100(±0)	91.7(±31.62)	0.92(±0.32)	0.958(±0.16)
	**DT**	87.5(±21.94)	100(±0)	75(±48.30)	0.75(±0.48)	0.84(±0.24)
	**RF**	87.5(±19.33)	91.7(±31.62)	83.3(±42.16)	0.75(±0.48)	0.941(±0)
**CFS**	**BN**	83.3(±0)	91.7(±0)	75(±0)	0.67(±0)	0.972(±0)
	**NB**	83.3(±15.81)	83.3(±0)	83.3(±31.62)	0.67(±0.32)	0.958(±0)
	**ANN**	87.5(±15.81)	91.7(±0)	83.3(±31.62)	0.75(±0.32)	0.972(±0)
	**SVM**	87.5(±15.81)	83.3(±0)	91.7(±31.62)	0.75(±0.32)	0.875(±0.16)
	**DT**	87.5(±18.00)	100(±0)	75(±42.16)	0.75(±0.42)	0.84(±0.21)
	**RF**	87.5(±21.94)	91.7(±31.62)	83.3(±42.16)	0.75(±0.48)	0.92(±0)
**Wrapper**	**BN**	70.8(±15.81)	66.7(±31.62)	75(±0)	0.42(±0.32)	0.859(±0)
	**NB**	83.3(±0)	83.3(±0)	83.3(±0)	0.67(±0)	0.955(±0)
	**ANN**	87.5(±10.54)	91.7(±0)	83.3(±31.62)	0.75(±0.32)	0.924(±0)
	**SVM**	95.8(±0)	91.7(±0)	100(±0)	0.92(±0)	0.958(±0)
	**DT**	79.2(±18.00)	83.3(±0)	75(±42.16)	0.58(±0.42)	0.799(±0.21)
	**RF**	66.7(±18.00)	58.3(±31.62)	75(±31.62)	0.33(±0.42)	0.729(±0.16)

The employment of the CFS algorithm maintains the following genes as most significant: A_24_P221960, THC2399272, BM683433, OXCT2, A_24_P230388, A_32_P57247 and AL566369. Next, the employment of the wrapper algorithm maintains the genes that are specifically tuned to achieve the best performance using a specific classification algorithm. Those genes are for BN: A_24_P221960, THC2399272, BM683433 and OXCT2; for NB: THC2410448, A_24_P221960, BM683433, OXCT2, A_32_P57247 and A_24_P942151; for ANN: A_24_P221960, THC2399272 and CN391963; for SVM: THC2410448, OXCT2 and A_24_P942151; for DT: A_24_P221960; for RF: A_24_P221960 and THC2399272.

#### Multi-type classifier

Next, we employ the best performing classification schemes identified using each source of data separately, and merge the individual predictions using a weighted majority voting algorithm, achieving perfect discrimination between patients with and without disease relapse. Table 10 shows the classification schemes based on each source of data.

**Table 10:** Best performing classifications schemes based on each source of data

**Source of data**	**Feature selection**	**Classifier**	**Acc (%)**	**Se (%)**	**Sp (%)**	**Kappa**	**AUC**
**Clinical**	Wrapper	DT	83.3 (±10.06)	73.7 (±14.35)	93 (±8.05)	0.67 (±0.20)	0.842 (±0.09)
**Imaging**	Wrapper	NB	90.9 (±12.25)	88.6 (±14.76)		93.2 (±14.15)	0.82 (±0.24)	0.89 (±0.12)
**Tissue genomic**	Union of genes from literature and current work	ANN	91.23 (±7.23)	94.7 (±11.25)		87.7 (±13.72)	0.82 (±0.14)	0.957 (±0.04)
**Blood genomic**	No feature selection	ANN	95.8 (±15.81)	100 (±0)		91.7 (±31.62)	0.92 (±0.32)	1 (±0)

### Disease evolution monitoring

For the second part of our analysis, we employ gene expression values extracted from blood samples which have been collected in predefined time-intervals during the follow-up period. After the raw values (45,015 genes) have been accordingly preprocessed, concluding in 33,491 high quality genes, we employ the SAM algorithm for time-course gene expression data in order to identify the genes that exhibit the most differential expression pattern over the follow-up. These retained genes (Table 11) are subsequently fed as input to the next steps of our analysis where we aim to monitor the disease evolvement.

**Table 11:** List of mostly differentially expressed genes over the follow-up period

**GeneID**	**Fold change**
HMCN1	2.5
RGMA	1.8
TSC1	2.2
AK023526	4.7
NOTCH2	2.8
STX6	4.8
THC2447689	2.9
THC2344152	1.9
LEPRE1	2.3

**Figure 5:**
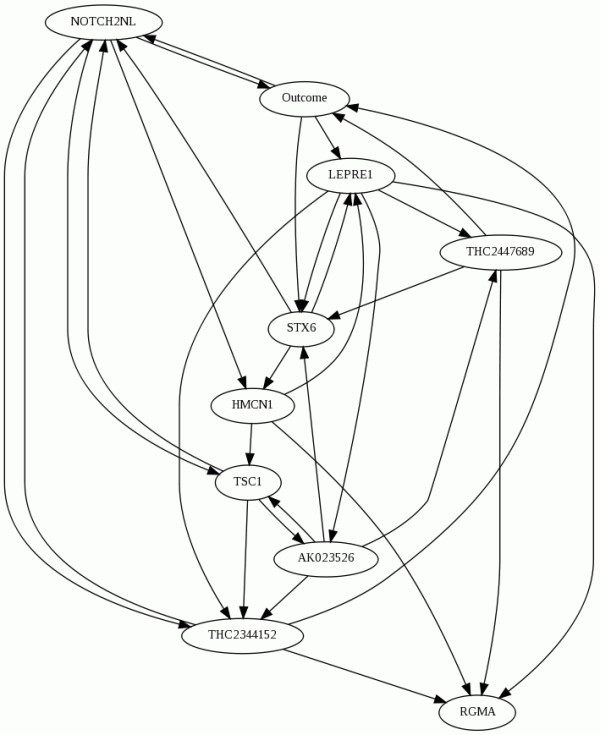
Best performing DBN architecture.

The aforementioned genes serve as input in order to formulate the architecture of the DBN, used subsequently for monitoring the evolvement of the disease over the follow-up. Specifically, we search among thousands of possible architectures using the Simulated Annealing and the Greedy algorithm in order to identify the best-performing ones, i.e. the ones yielding the highest results. It should be noted that no restrictions have been imposed on the nodes of the network, in order to obtain the network whose interactions yield the highest performance. In Figure 5 we present the DBN architecture that yielded the highest results.

For the evaluation of the trained DBN model, we have employed the leave-one-patient out technique, and based on the individual predictions we have calculated the overall results, that are shown in Table 12. According to the employed clinical scenario, gene expression from blood samples is extracted in three consecutive visits, that refer to the baseline visit, the middle of the follow-up period (i.e. follow-up #1) and the end of the 2-year follow-up (follow-up #2). In Table 12, the first row shows the performance of the DBN model towards the relapse probability at follow-up #1, using as input solely the data from the baseline; the second row shows the performance towards predicting the relapse probability at follow-up #2, using as input data both from the baseline visit and follow-up #1.

**Table 12:** Overall performance of the DBN model

**Evidence**	**Sensitivity (%)**	**Specificity (%)**	**Accuracy (%)**
**Baseline**	63.6	100	86
**Baseline & follow-up #1**	100	100	100

## Discussion

In this work we have collected and analyzed a broad set of heterogeneous data from various sources, i.e. clinical, imaging tissue genomic and blood genomic, in order to capture the progression of the disease during remission and predict potential disease relapses. For this purpose a twofold analysis has been performed: i) Baseline Data Analysis which employs solely data obtained at the baseline visit aiming to identify a potential disease relapse and ii) Disease Evolution Monitoring, that explores gene expression from genes obtained at predefined intervals over the follow-up coupled with a personalized genetic signature in order to capture the temporal progression of the disease and hence predict the approximate timing of a potential relapse.

The Baseline Data Analysis outcome is compared with the works presented in the literature review, as shown in Table 13.

**Table 13:** Comparison between the current work and the literature

**Author**	**Number of patients**	**Accuracy (%)**
Roepman et al. [7]	22	86
Roepman et al. [8]	66	88
Rickman et al. [9]	79	77
Watanabe et al. [10]	39	76
Nagata et al. [11]	75	87
Zhou et al. [12]	25	85
**Exarchos et al. (current work)**	**86**	**100**

We observe that the currently proposed methodology exhibits superior results compared to the other methods presented in the literature, nevertheless, direct comparison cannot be performed since different datasets have been employed in each case. It should be noted that the currently employed dataset contains a considerable number of patients, i.e. 86, compared to the other ones in the literature. A significant advantage of the current work is the multitude of data that has been employed (clinical, imaging, tissue genomic and blood genomic) whereby a separate classifier has been trained from each source of data, as well as an overall one that combines the individual classifiers. On the other hand, the other methodologies in the literature exploit mostly genomic data coupled with information from the clinical profile of the patients.

In addition, the modular architecture of the current work allows for inspecting the "verdict" based on each source of data separately and hence conjecturing about the contribution and validity of each type of data. Of course an overall decision is calculated which weighs the individual predictions yielding a more accurate consensus outcome. Alternatively, the combination of all sources of data could be achieved by pooling all data in a single dataset (i.e. the bag of features approach); however, with this approach the intersection of patients across all sources of data would have to be used, and given the uneven distribution of patients this would conclude in a rather limited dataset. In terms of validation, the current work has been evaluated using 10-fold cross validation and the leave-one-patient-out technique, in order to assess thoroughly the achieved performance. The results obtained with the leave-one-patient-out technique are in complete accordance with the ones obtained using 10-fold cross validation, therefore, the former ones are not included in the manuscript.

The best performing classification schemes based on each source of data are summarized in Table 10. The features maintained in each case constitute a minimal subset of features that bears enhanced discriminative potential. Therefore, in terms of prediction, we have come down to a rather refined set of features that can be employed in order to estimate the relapse probability for a specific patient.

The features maintained from each source of data can be inspected independently, however, it should be noted that it is their combination that yields the results presented previously. Based on the clinical data, the best performing classification scheme involves a Decision Tree where the initial input has been stripped off from redundant features using the wrapper feature selection algorithm, maintaining the following features as most significant: depth of invasion, p16ink4a stain and N staging. Especially the depth of invasion and the N staging constitute major factors affecting the disease prognosis. For the case of imaging data the Naive Bayes classifier coupled with the wrapper algorithm for feature selection employs the combination of extra-tumor spreading and (lymph node) site, thus, achieving the highest performance. Next, we move on to the genomic data (from tissue and blood) where the best performing gene subsets are shown in Table 5 and Table 8, respectively. Among those genes, there are certain genes that have been correlated in the literature with the evolvement of oral cancer and its manifestations. Specifically, TCAM1 has been associated with the HPV status of patients with head and neck squamous cell carcinomas [25]; another example is SOD2 that has been implicated with the progression and metastasis of oral cancer [26,27]. For the remaining genes more distant and sporadic associations have been mined in the literature for relevant diseases or other types of cancer, e.g. AMDHD1 and PHACTR1 have been linked to tobacco use disorders [28] or RPRM with colorectal cancers [29].

Both for the clinical and the imaging input vector, the employment of the wrapper algorithm yields the feature subset with the highest discriminating ability; in accordance with our findings, the wrapper algorithm has been reported in the literature to often outperform other feature selection algorithms due to the fact that it is tuned to the target classification algorithm [19]. However, in the case of tissue and blood genomic data, the output from the SAM algorithm constitutes the best performing gene subset, and further feature selection does not ameliorate the results; this is more or less expected since the SAM algorithm aims at finding those genes that differ the most between the two target classes, and therefore bear enhanced discriminating potential.

An important aspect of the current work that should be highlighted, is the fact that for the tissue genomic data, we have merged the gene subset identified using the current dataset with a set of genes pinpointed in the literature as highly correlated and descriptive of oral cancer reoccurrence. As shown in the Results section, the union of the two gene subsets achieves superior performance compared to the individual sets. Besides the performance amelioration, it is very important that in this manner we consolidate information from other data resources, thus, achieving enhanced generalization capability.

As for the Disease Evolution Monitoring, a substantial advancement is the incorporation of the time dimension, thus, capturing the temporal nature of the disease. This is particularly interesting from a medical point of view, since we are able to conjecture the approximate timing that a potential relapse is more likely to occur. Moreover, the ability of DBNs to capture time-varying parameters, resembles better the actual disease progression and facilitates the modeling of the evolvement over the follow-up period. The employment of a DBN which features a transparent architecture allows for inspecting the interplay among the involved parameters and therefore, reasoning is provided for each decision. DBNs have been elsewhere used in other domains in order to capture time-varying events, yielding quite satisfactory and accurate results.

As shown in the DBN architecture depicted in Figure 5, nine genes have been maintained as most discriminatory and their combination, as represented in the DBN dependencies, can be used to conjecture about the relapse probability of a specific patient. The genes that have been maintained are: HMCN1, RGMA, TSC1, AK023526, NOTCH2, STX6, THC2447689, THC2344152, LEPRE1. It should be noted that the features extracted from the personalized genetic signature that has been described previously, were not maintained during the training of the DBN and therefore they have not been included in the employed architecture. The majority of the aforementioned genes have not been elsewhere associated in the literature with oral cancer progression; however, gene TSC1 is a notable exception since it has been correlated with esophageal cancer as well as the reoccurrence of head and neck cancer [30,31]. Furthermore, gene AK023526 has been found to constitute a marker for cancer stem cells [32], and gene NOTCH2 has been associated with tobacco use disorders and certain types of cancer [28].

The relatively small association of the extracted genes with literature findings related to cancer and more specifically oral cancer, was expected since blood gene expression has been scarcely studied, and therefore few and sporadic references exist in the literature. However, given the fact that even a subset of the currently extracted genes have been identified in cancer related pathways, is quite encouraging and further supports the proof-of-concept that is intended with the approach of Disease Evolution Monitoring in the current work. In any case, the relatively small set of patients, compared to the initially enormous number of genes was a hindrance in the first place, yet the preliminary results obtained using the leave-one-patient-out technique are quite satisfactory.

## Conclusions

In this work we have presented a multiscale and multiparametric approach for modeling the progression of oral cancer during remission. Specifically, our approach consist of two main analyses, i) Baseline Data Analysis where clinical, imaging, tissue genomic and blood genomic data were employed in order to predict a potential disease relapse and ii) Disease Evolution Monitoring aiming to capture the progression of the disease based on gene expression data acquired from circulating blood cells, and subsequently exploit this information in order to predict if and roughly when a relapse is more likely to appear. This information can be used to stratify the patients into high and low risk groups according to the relapse probability; hence, the treatment protocol can be either intensified or relaxed accordingly. Moreover, it is very important to unify our study with findings from similar studies alongside with further verification of the results in order to achieve enhanced generalization capability and conjecture meaningful and solidified corollaries.

The proposed approach encompasses heterogeneous sources of data that are expected to assess the disease status from several aspects and therefore, can be proven very helpful towards studying complex diseases, such as cancer.

## Competing interests

The authors declare that they have no competing interests.

## Authors’ contributions

KPE and YG conceived, designed and implemented the study, DIF supervised the study and provided substantial advice and guidance during all phases. All authors have read and approved the final manuscript.

## Pre-publication history

The pre-publication history for this paper can be accessed here:

http://www.biomedcentral.com/1472-6947/12/136/prepub
